# The Relationship Between the Frequency of Hair Shampooing and the Condition of Hair and Scalp Among the Population of Saudi Arabia

**DOI:** 10.1155/drp/3472173

**Published:** 2026-05-24

**Authors:** Nouf Bin Rubaian, Fawzeyah Alnakhli, Lama M. Albelowi, Asrar Alharbi, Danah Alnajjar, Deemah S. AlHuraish

**Affiliations:** ^1^ Dermatology Department, Imam Abdulrahman Bin Faisal University (IAU), King Fahad Hospital of the University (KFHU), Al-Khobar, Eastern Province, Saudi Arabia, iau.edu.sa; ^2^ College of Medicine, Imam Abdulrahman Bin Faisal University, Dammam, Eastern Province, Saudi Arabia, iau.edu.sa; ^3^ Dermatology Department, King Salman Bin Abdulaziz Medical City, Medina, Saudi Arabia; ^4^ College of Medicine, Taibah University, Al-Madinah Al-monawarraah, Western Province, Saudi Arabia, taibahu.edu.sa

**Keywords:** care products, dermatological study, hair health, hair loss, hair wash, scalp health

## Abstract

**Objective:**

The appearance of our physical features, including our hair, holds great significance in our lives. Shampooing is a widely adopted practice for improving hair condition. However, there is an ongoing debate regarding the impact of shampooing frequency on hair and scalp health. This study aimed to investigate the relationship between the frequency of hair shampooing and the condition of both hair and scalp.

**Methods:**

A cross‐sectional study design was employed to gather data through online survey questionnaires administered in Arabic and targeted at Saudi Arabian citizens. The study included a sample size of 4796 participants from various clinics across Saudi Arabia. Descriptive statistics were utilized to analyze the data, calculating frequencies and percentages for different groups. Chi‐square tests and inferential analysis were conducted to determine the association between variables.

**Results:**

The study identified a significant correlation between hair type and hair problems. Long hair and hairstyles with curls were associated with higher rates of hair breakage and daily hair loss. The findings demonstrated that the frequency of hair shampooing directly influenced the condition of both hair and scalp. Increased shampooing frequency was found to improve hair and scalp conditions.

**Conclusion:**

In conclusion, this study establishes that a higher frequency of shampooing enhances the condition of both hair and scalp. Therefore, we recommend that individuals aiming to enhance their physical appearance should consider increasing the frequency of shampoo usage.

## 1. Introduction

The condition of the hair plays a significant role in our physical appearance and self‐perception, and this is true for many people around the world, including the residents of Saudi Arabia. The quality, quantity, and styling of one’s hair can define aspects such as gender, age, health, and social status [1]. It is important to note that while hair can differentiate individuals physically, there is no notable difference in the number of hair follicles based on races, gender, or social classes.

The condition of both hair and the scalp can vary from person to person due to various factors, including the type of hair treatment they used, diet, hormonal changes, stress, and the overall health. While some individuals may have healthy hair and scalp, others may experience a range of issues concerning their hair and scalp health. A healthy scalp is characterized by clean, well‐hydrated skin, good blood circulation, and balanced oil production [1, 2]. Having a healthy scalp, devoid of inflammation, itching, and flaking, is indicative of good overall condition. Likewise, well‐maintained hair exhibits traits such as softness, strong elasticity, the capacity for substantial growth, and a pleasing texture. Additionally, hair that remains intact and does not experience excessive breakage or shedding is also considered to be in a good condition [3–5]. However, the difference in appearance between people is also attributed to the type of hair produced by the follicle and the type of routine scalp and hair care practiced by individuals. A vital aspect of hair care is the frequency at which individuals shampoo their hair, as it directly impacts the condition of both the hair strands and the scalp.

Despite the widespread use of shampoo in daily hair care routines, the optimal frequency of shampooing and its impact on hair and scalp health remains a subject of debate. Limited data are available regarding shampooing practices and their dermatological implications among the Saudi population.

Therefore, this study aimed to investigate the association between the frequency of hair shampooing and the condition of hair and scalp among the population of Saudi Arabia.

## 2. Materials and Methods

### 2.1. Study Design and Population

The study utilized a cross‐sectional design to collect data from different groups of respondents simultaneously. Participants were categorized into two groups: low‐frequency shampooers and high‐frequency shampooers, and they were administered the same survey questionnaire. The cross‐sectional design was chosen to enable data collection from both groups during the same time frame, under similar conditions, facilitating the comparison of their characteristics. The study was conducted across all regions of the Kingdom of Saudi Arabia, aiming to include all eligible citizens and residents as the study population. The sample size of 664 participants was determined using the Raosoft calculator, with a 99% confidence interval and a 5% margin of error. Convenience sampling was employed to draw the sample, including eligible citizens within the Kingdom, and participants meeting the inclusion criteria were invited to complete the online questionnaire until the desired sample size was achieved.

The minimum required sample size was calculated using the Raosoft sample size calculator assuming a 99% confidence level, 5% margin of error, and an estimated response distribution of 50%. The calculated minimum sample size was 664 participants. However, a larger number of participants (*n* = 4796) completed the questionnaire and were included in the final analysis.

### 2.2. Survey Methodology and Data Analysis

Data collection techniques and tools included the development of an online questionnaire in both English and Arabic languages, ensuring accessibility for all Saudi citizens and maximizing the reach to eligible participants. The questionnaire was divided into three sections: the first section comprised a consent form for participants to sign, indicating their willingness to participate in the study. The second section collected demographic and social information, while the third section focused on shampooing habits and hair and scalp conditions. Data collection was conducted online through the use of a questionnaire that had undergone expert review. The questionnaire was made accessible to participants through a provided Uniform Resource Locator (URL) on Google Forms. Participants meeting the inclusion criteria, including willingness to participate, Saudi Arabian citizenship or residency, age 18 years or older, and completion of the questionnaire, were invited to take part. The primary independent variable in this study was the frequency of shampoo use per week. Dependent variables included reported hair loss, dandruff, and hair breakage. Additional variables included hair type, hair length, scalp type, use of hair conditioner, use of hairstyling products, and presence of medical conditions affecting hair. Prior to the main data collection, a pilot study was conducted with 30 individuals to assess the validity and feasibility of the tools, and the obtained data from the pilot study were excluded from the final dataset. Statistical analysis was performed using IBM SPSS Statistics Version 23 (IBM Corp., Armonk, NY, USA). Descriptive statistics were used to summarize demographic characteristics and hair‐related variables, and the results were presented as frequencies and percentages. Associations between categorical variables were assessed using the Chi‐square test. A *p* value < 0.05 was considered statistically significant.

#### 2.2.1. Ethical Considerations

The study received ethical approval from the Institutional Review Board (IRB) of Imam Abdulrahman Bin Faisal University (IRB‐2024‐01‐008), ensuring adherence to ethical standards. Informed consent was obtained from all participants, who were fully informed about the purpose of the study and assured of their voluntary participation. Participation in the study was voluntary, and participants could withdraw at any time without consequences. Confidentiality and anonymity were strictly maintained throughout the study to protect the privacy of the participants. Additionally, participant well‐being was prioritized throughout the research process to ensure a safe and respectful environment.

## 3. Results

The study collected the data from 4796 participants. The age distribution of the participants revealed that the majority fell within the 18–22‐year old category (*n* = 1864), while the lowest number of participants was the 28–34‐year old category (*n* = 651). Similarly, the proportion of females 77.7% was higher than that of males 22.3%. Regarding hair and scalp data, most of the participants had shoulder‐length hair (45.8%), while most of them had wavy hair (54.7%), and the type of scalp was oily among 59.6% of the participants (Table 1).

**TABLE 1:** Descriptive statistics of the study participants.

Age	Frequency	Percent
18–22	1864	38.9
23–27	1067	22.2
28–34	651	13.6
≥ 35	1214	25.3
Gender		
Male	1069	22.3
Female	3727	77.7
Hair length		
Close cut	500	10.4
Ear length	602	12.6
Between ear and shoulder	489	10.2
Shoulder length	1009	21.0
Below the shoulder	2196	45.8
Hair type		
Straight	1243	25.9
Wavy	2623	54.7
Curly	930	19.4
Scalp type		
Oily	2858	59.6
Dry	1938	40.4

Hair loss problem among the study participants was analyzed with the hair length, type, and scalp type. It was found that a significantly high proportion of the participants who had below shoulder‐length hairs was suffering from hair loss (*p* ≤ 0.001). Similarly, those who had wavy hairs showed a significantly high proportion (77.2%), having hair loss problems (*p* ≤ 0.001). In addition, those who had oily scalps showed a significantly high proportion (77.4%), who had hair loss problems (*p* ≤ 0.001) (Table 2).

**TABLE 2:** Association between hair and scalp type with hair loss problem.

Hair length	Suffer from hair loss		*p* value
	Yes	No	
Close cut	287 (57.4)	213 (42.6)	≤ 0.001^∗^
Ear length	333 (55.3)	269 (44.7)	
Between ear and shoulder	359 (73.4)	130 (26.6)	
Shoulder length	837 (83.0)	172 (17.0)	
Below the shoulder	1796 (81.8)	400 (18.2)	
Hair type			
Straight	883 (71.0)	360 (29.0)	≤ 0.001^∗^
Wavy	2026 (77.2)	597 (22.8)	
Curly	703 (75.6)	227 (24.4)	
Scalp type			
Oily	2211 (77.4)	647 (22.6)	≤ 0.001^∗^
Dry	1401 (72.3)	537 (27.7)	

^∗^Statistically significant at 0.05 level of significance.

The presence of dandruff was found to be significantly associated with certain factors related to hair length, type, and scalp type. Specifically, individuals with close‐cut hair had a significantly higher proportion (51.2%) of experiencing dandruff problems (*p* = 0.026). Similarly, dandruff was found to be more common in individuals with curly hair compared to those with straight or wavy hair (*p* ≤ 0.001). Additionally, individuals with oily scalps were found to have a higher frequency of dandruff problems compared to those with dry scalps (*p* ≤ 0.001) (Table 3).

**TABLE 3:** Association between hair and scalp type with dandruff problem.

Hair length	Suffer from dandruff		*p* value
	Yes	No	
Close cut	256 (51.2)	244 (48.8)	0.026**^∗^**
Ear length	291 (48.3)	311 (51.7)	
Between ear and shoulder	203 (41.5)	286 (58.5)	
Shoulder length	455 (45.1)	554 (54.9)	
Blew the shoulder	1007 (45.9)	1189 (54.1)	
Hair type			
Straight	540 (43.4)	703 (56.6)	≤ 0.001^∗^
Wavy	1188 (45.3)	1435 (54.7)	
Curly	484 (52.0)	446 (48.0)	
Scalp type			
Oily	1445 (50.6)	1413 (49.4)	≤ 0.001^∗^
Dry	767 (39.6)	1171 (60.4)	

*Note:* The bold values indicate statistically significant *p* values (*p* < 0.05). These are 0.026 for hair length and ≤ 0.001 for hair type and scalp type.

^∗^Statistically significant at 0.05 level of significance.

An analysis of hair length and its association with hair breakage revealed that as hair length increased, the incidence of hair breakage also increased. Specifically, individuals with hair length below the shoulder had a significantly higher proportion experiencing hair breakage compared to those with shorter hair length (*p* ≤ 0.001). Similarly, individuals with curly hair were found to have a significantly higher proportion experiencing hair breakage problems (*p* ≤ 0.001). Additionally, a significant association was observed between scalp type and hair breakage, with dry scalps being more commonly associated with hair breakage compared to oily scalps (*p* = 0.017) (Table 4).

**TABLE 4:** Association between hair and scalp type with hair breakage problem.

Hair length	Suffer from hair breakage		*p* value
	Yes	No	
Close cut	189 (37.8)	311 (62.2)	≤ 0.001^∗^
Ear length	239 (39.7)	363 (60.3)	
Between ear and shoulder	279 (57.1)	210 (42.9)	
Shoulder length	695 (68.9)	314 (31.1)	
Blew the shoulder	1616 (73.6)	580 (26.4)	
Hair type			
Straight	623 (50.1)	620 (49.9)	≤ 0.001^∗^
Wavy	1748 (66.6)	875 (33.4)	
Curly	647 (69.6)	283 (30.4)	
Scalp type			
Oily	1759 (61.5)	1099 (38.5)	0.017**^∗^**
Dry	1259 (65.0)	679 (35.0)	

Note: The bold values indicate statistically significant *p* values (*p* < 0.05). These are ≤ 0.001 for hair length and hair type, and 0.017 for scalp type.

^∗^Statistically significant at 0.05 level of significance.

The effect of hair length, type, and scalp type on hair loss was analyzed and presented in Table 5. The analysis revealed a significant correlation between hair length and daily hair loss, with an increase in hair length associated with a higher rate of hair loss per day (*p* ≤ 0.001). Similarly, individuals with straight hair exhibited significantly less daily hair loss compared to those with wavy or curly hair (*p* ≤ 0.001).

**TABLE 5:** Association between hair and scalp type with per day hair loss.

Hair length	Hair loss per day				*p* value
	> 50	100–150	150–300	> 300	
Close cut	343 (68.6)	103 (20.6)	32 (6.4)	22 (4.4)	≤ 0.001^∗^
Ear length	373 (62.0)	172 (29.1)	40 (6.6)	14 (2.3)	
Between ear and shoulder	209 (42.7)	198 (40.5)	66 (13.5)	16 (3.3)	
Shoulder length	339 (33.6)	461 (45.7)	162 (16.1)	47 (4.7)	
Blew the shoulder	734 (33.4)	1079 (49.1)	305 (13.6)	78 (3.6)	
Hair type					
Straight	625 (50.3)	447 (36.0)	130 (10.5)	41 (3.3)	≤ 0.001^∗^
Wavy	1002 (38.2)	1165 (44.4)	355 (13.5)	101 (3.9)	
Curly	371 (39.9)	404 (43.4)	120 (12.9)	35 (3.8)	
Scalp type					
Oily	1201 (42.0)	1198 (41.9)	357 (12.5)	102 (3.6)	**0.893**
Dry	797 (41.1)	818 (42.2)	248 (12.8)	75 (3.9)	

*Note:* The bold values indicate statistically significant *p* values (*p* < 0.05). These are ≤ 0.001 for hair length and hair type, and 0.893 for scalp type.

^∗^Statistically significant at 0.05 level of significance.

The relationship between hair breakage, hair loss, and medical conditions that can affect the hair or scalp is summarized in Table 6. The analysis revealed that a significant proportion of participants (74.5%) with medical conditions experienced hair breakage problems, while only 25.5% of participants with medical conditions did not experience hair breakage. This difference in proportions was found to be statistically significant (*p* ≤ 0.001).

**TABLE 6:** Association between medical condition with hair breakage and hair loss.

Suffer from hair breakage	Medical condition affect the hairs		*p* value
	Yes	No	
Yes	570 (74.5)	2448 (60.7)	≤ 0.001^∗^
No	195 (25.5)	1583 (39.3)	
Hair loss per day			
> 50	284 (37.1)	1714 (42.5)	≤ 0.001^∗^
100–150	299 (39.1)	1717 (42.6)	
150–300	142 (18.6)	463 (11.5)	
> 300	40 (5.2)	137 (3.4)	

^∗^Statistically significant at 0.05 level of significance.

Furthermore, the analysis of “per day hair loss in participants with medical conditions” indicated that 39.1% of participants with medical conditions experienced a hair loss range of 100–150 hairs per day. This percentage was significantly higher compared to participants who experienced a hair loss range of 150–300 hairs per day or more than 300 hairs per day (*p* ≤ 0.001).

When examining the frequency of shampoo use per week in relation to hair breakage and hair loss, it was observed (Table 7) that individuals who used shampoo daily experienced the least hair breakage problems, while those who used shampoo up to 3 times per week had the highest incidence of hair breakage (*p* ≤ 0.001). Similarly, it was found (Table 7) that individuals who shampooed their hair daily had minimal hair loss per day (< 50), whereas those who used shampoo less frequently experienced higher rates of hair loss (*p* ≤ 0.001).

**TABLE 7:** Frequency of shampoo use per week in association with hair breakage and hair loss.

Suffer from hair breakage	Number of times use shampoo per week				*p* value
	1	2‐3	4‐5	Daily	
Yes	308 (65.7)	1677 (66.7)	710 (60.6)	323 (50.2)	≤ 0.001^∗^
No	161 (34.3)	836 (33.3)	461 (39.4)	320 (49.8)	
Hair loss per day					
> 50	221 (47.1)	940 (37.4)	499 (42.6)	338 (52.6)	≤ 0.001^∗^
100–150	178 (38.0)	1188 (47.3)	459 (39.2)	191 (29.7)	
150–300	48 (10.2)	314 (12.5)	164 (14.0)	79 (12.3)	
> 300	22 (4.7)	71 (2.8)	49 (4.2)	35 (5.4)	

^∗^Statistically significant at 0.05 level of significance.

The effect of the use of hair conditioner on hair breakage and per day hair loss provided that over 70% of the participants were using the conditioner and suffering from hair breakage problems (*p* ≤ 0.001). In addition, the use of conditioner causes an increase in per day hair loss compared to those who did use the conditioners (Table 8).

**TABLE 8:** Effect of use of conditioner in association with hair breakage and hair loss.

Suffer from hair breakage	Do you use hair conditioner		*p* value
	Yes	No	
Yes	1742 (70.3)	1276 (55.0)	≤ 0.001^∗^
No	735 (29.7)	1043 (45.0)	
Hair loss per day			
> 50	931 (37.6)	1067 (46.0)	≤ 0.001^∗^
100–150	1107 (44.7)	909 (39.2)	
150–300	353 (14.3)	252 (10.9)	
> 300	86 (3.5)	91 (3.9)	

^∗^Statistically significant at 0.05 level of significance.

Similarly, the impact of hairstyle products on hair breakage and daily hair loss was examined. The study revealed a significant association between the use of hair styling products and hair breakage problems, with 73.0% of individuals using these products experiencing hair breakage (*p* ≤ 0.001) (Table 9). Furthermore, the frequency of daily hair loss was found to significantly increase among those using hair styling products (*p* ≤ 0.001) (Table 9).

**TABLE 9:** Effect of use of hair style products in association with hair breakage and hair loss.

Suffer from hair breakage	Do you use hair style products		*p* value
	Yes	No	
Yes	1635 (73.0)	1383 (54.1)	≤ 0.001^∗^
No	605 (27.0)	1173 (45.9)	
Hair loss per day			
> 50	859 (38.3)	1139 (44.6)	≤ 0.001^∗^
100–150	986 (44.0)	1030 (40.3)	
150–300	318 (14.2)	287 (11.2)	
> 300	77 (3.4)	100 (3.9)	

^∗^Statistically significant at 0.05 level of significance.

The relationship between the frequency of shampoo usage, the use of hair conditioner, and hair loss was examined in relation to daily hair loss. The study revealed that individuals who reported using conditioner and experiencing hair loss had a higher daily hair loss (> 300) compared to those who reported negatively or lacked knowledge on the subject (*p* = 0.001). Additionally, the perception of certain shampoos promoting hair growth was found to be associated with daily hair loss. Those who believed that certain shampoos could enhance hair growth exhibited significantly lower levels of hair loss per day (*p* ≤ 0.001) (Table 10).

**TABLE 10:** Association of perception of hair loss and growth with the number of hair loss per day.

Frequent shampooing causes hair loss	Hair loss per day				*p* value
	> 50	100–150	150–300	> 300	
Yes	913 (40.1)	1003 (44.0)	286 (12.6)	76 (3.3)	**0.154**
No	438 (44.2)	389 (39.2)	123 (12.4)	42 (4.2)	
I don’t know	647 (42.4)	624 (40.9)	196 (12.8)	59 (3.9)	
Conditioner causes hair loss					
Yes	567 (39.3)	603 (41.8)	206 (14.3)	65 (4.5)	0.001**^∗^**
No	701 (40.5)	772 (44.6)	209 (12.1)	48 (2.8)	
I don’t know	730 (44.9)	641 (39.4)	190 (11.7)	64 (3.9)	
Certain shampoo helps in hair growth					
Yes	939 (43.2)	904 (41.6)	267 (12.3)	62 (2.9)	≤ 0.001^∗^
No	583 (40.1)	651 (44.8)	171 (11.8)	49 (3.4)	
I don’t know	476 (40.7)	461 (39.4)	167 (14.3)	66 (5.6)	

*Note:* The bold values indicate statistically significant *p* values (*p* < 0.05). These are 0.001 for conditioner causes hair loss and ≤ 0.001 for certain shampoo helps in hair growth. The value 0.154 for frequent shampooing causes hair loss is not statistically significant (*p* > 0.05).

^∗^Statistically significant at 0.05 level of significance.

## 4. Discussion

Hair and scalp health are influenced by a complex interaction of genetic, environmental, and behavioral factors. Hair care practices, including the frequency of shampooing, use of conditioners, and styling habits, may affect scalp physiology and the structural integrity of hair fibers. Understanding these relationships is important for dermatologists when advising patients on evidence‐based hair care practices. The present study aimed to investigate the association between shampooing frequency and the condition of hair and scalp among the population of Saudi Arabia.

Our findings demonstrated that hair characteristics, including hair length and hair type, were significantly associated with reported hair problem. Participants with longer hair reported higher rates of hair loss and hair breakage compared with those with shorter hair. Previous studies have suggested that longer hair may be more susceptible to mechanical stress and environmental damage due to increased exposure and repeated grooming practices [5, 6]. Repeated mechanical manipulation of long hair, including brushing, styling, and environmental exposure, may weaken the hair shaft and increase the likelihood of hair fragility and breakage [7]. Additionally, cosmetic treatments and environmental factors can alter the lipid and protein composition of the hair shaft, further contributing to structural damage [3, 8].

Hair morphology also appeared to influence the prevalence of hair problems in our study population. Participants with wavy and curly hair reported higher rates of hair breakage and hair loss compared with individuals with straight hair. This finding is consistent with previous dermatological research indicating that hair fiber curvature can influence the distribution of mechanical forces along the hair shaft [9, 10]. Curly and wavy hair fibers often have irregular cross‐sectional structures, which may predispose them to increased fragility and susceptibility to cosmetic and environmental damage.

Scalp characteristics also played a significant role in hair and scalp conditions in this study. Participants with oily scalps reported higher frequencies of dandruff and hair loss compared with those with dry scalps. Excess sebum production may promote the proliferation of *Malassezia* species, which are recognized contributors to dandruff and seborrheic dermatitis [11]. The interaction between sebaceous lipids, scalp microbiota, and individual inflammatory responses may result in scalp irritation, flaking, and altered hair growth dynamics [12–14].

One of the most notable findings of this study was the association between shampooing frequency and hair and scalp condition. Participants who reported washing their hair more frequently, particularly on a daily basis, tended to report lower rates of hair breakage and lower levels of daily hair loss compared with those who washed their hair less frequently. Regular cleansing of the scalp may help remove excess sebum, sweat, environmental pollutants, and microbial accumulation that can negatively affect scalp health [6, 15]. Proper cleansing may also help maintain the integrity of the hair cuticle by preventing the accumulation of irritants and oxidative by‐products on the scalp surface [2, 16].

Previous dermatologic studies have similarly suggested that frequent shampooing does not necessarily increase hair loss and may contribute to improved scalp hygiene and healthier scalp environments [17]. Maintaining scalp cleanliness may reduce oxidative stress and inflammatory processes that could affect hair follicle function and hair retention [2]. In addition, modern shampoo formulations are designed to effectively cleanse the scalp while minimizing damage to the hair shaft when used appropriately [18, 19].

Interestingly, our study also found that participants who reported using hair conditioners and styling products had higher rates of reported hair breakage and hair loss. While conditioners are commonly used to improve hair manageability and reduce friction between hair fibers, certain formulations may contribute to hair shaft weakening if used excessively or if they contain harsh chemical agents [3]. Cosmetic hair products can alter the lipid composition of the hair cuticle and affect the mechanical strength of hair fibers, potentially increasing susceptibility to breakage. Furthermore, the buildup of styling products on the scalp and hair shaft may contribute to scalp irritation or hair fragility if not adequately removed during cleansing [20].

The findings of this study contribute to the growing body of literature examining the dermatological implications of hair care practices in different populations. In regions such as Saudi Arabia, environmental conditions including high temperatures, dust exposure, and humidity fluctuations may influence scalp physiology and hair care behaviors [21, 22]. Understanding these environmental and cultural factors is important for dermatologists when providing patient education regarding optimal hair hygiene and scalp care practices.

Several limitations should be considered when interpreting the findings of this study. First, the study relied on self‐reported data, which may introduce recall bias or subjective misclassification of hair conditions. Second, the cross‐sectional design limits the ability to establish causal relationships between shampooing frequency and hair health outcomes. Future studies incorporating objective dermatologic assessments, such as trichoscopic evaluation or clinical scalp examinations, may provide more accurate evaluation of hair and scalp conditions. Longitudinal studies may also help clarify the long‐term effects of different hair care practices on hair health.

Despite these limitations, the study provides valuable insights into hair care practices among a large sample of the Saudi population. The large sample size and nationwide distribution of participants enhance the generalizability of the findings. These results highlight the potential importance of scalp hygiene and appropriate shampoo use in maintaining healthy hair and scalp conditions.

## 5. Conclusion

This large cross‐sectional study provides valuable insights into hair care practices and their association with hair and scalp conditions among the Saudi population. Higher shampooing frequency was associated with lower reported rates of hair breakage and daily hair loss, suggesting that regular scalp cleansing may contribute to improved scalp hygiene and hair condition. These findings may help guide dermatologic counseling on hair care practices, although further studies using objective clinical assessments are needed to confirm these associations.

**Nomenclature**
IRB: Institutional Review Board
URL: Uniform Resource Locator
IBM SPSS: International Business Machines Corporation Statistical Package for the Social Sciences

## Funding

All authors have declared that they have no financial relationships at the present or within the previous three years with any organizations that might have an interest in the submitted work.

## Conflicts of Interest

The authors declare no conflicts of interest.

## Data Availability

The data that support the findings of this study are available on request from the corresponding author. The data are not publicly available due to privacy or ethical restrictions.

## Appendix A

Questionnaire

1. What is your gender? Male/Female
2. How old are you? 18–22/23–27/28–34/> 35

Section 2

1. What is the length of your hair? Close cut/Ear length/Between ear and shoulder/Shoulder length/Below the shoulder
2. What is your hair type? Straight/Wavy/Curly
3. What is your scalp type? Oily/Dry
4. How often do you wash your hair per week? 1/2‐3/4‐5/daily
5. Do you use hair conditioner? Yes/No
6. Do you use any hair styling products? Yes/No
7. Do you suffer from dandruff? Yes/No
8. Do you suffer from hair breakage? Yes/No
9. Have you been diagnosed with any medical conditions related to your hair or scalp?
10. What is the normal hair loss count per day? < 50/100–150/150–300/> 300
11. Do you think shampooing your hair more than 4 times weekly causes hair loss? Yes/No/I don’t know
12. Do you think using hair conditioner causes hair loss? Yes/No/I don’t know
13. Do you believe that certain shampoos promote hair growth? Yes/No/I don’t know
